# Comparative analysis of growth cycles among three weedy *Avena* species: Insights from field observations

**DOI:** 10.1371/journal.pone.0307875

**Published:** 2024-09-13

**Authors:** Süleyman Gürdal Türkseven

**Affiliations:** Department of Plant Protection, Faculty of Agriculture, Ege University, Bornova, İzmir, Turkey; Abdul Wali Khan University Mardan, PAKISTAN

## Abstract

*Avena* species, especially *A*. *fatua*, *A*. *sterilis* and *A*. *ludoviciana*, are among the most problematic weed species in many crops worldwide. The growth cycles of these three species could be helpful in understanding their growth cycle and their implications for agriculture and weed management. The growth cycles of these species were studied altogether or in combination with any single or double combinations of the other species in cereal fields in Türkiye, using two populations of each in a common garden experiment in Bornova district, Izmir, Türkiye. Germination and growth experiments were conducted in the laboratory and screen house, respectively. Various phenological parameters were recorded during the experiment and data were analyzed using R software. There were no significant differences in germination, emergence, SPAD values, leaf width, plant height, or plant dry weight among the species or populations. The SPAD values and width of the flag leaf and the leaf before the flag leaf were strongly correlated. Plant weight increased with increasing tiller number. The length of the ligule in a population of *A*. *sterilis* was significantly greater than that in populations of two other species, and it was concluded that the species is not *A*. *fatua* or *A*. *ludoviciana* if the length of the ligule is greater than 10 mm. The length of the spikelets of *A*. *sterilis* was greater than 65 mm with awn and greater than 35 mm without awn; these values were significantly greater than those of two other species that were shorter than 55 with awn for *A*. *fatua* and 30 mm without awn for *A*. *ludoviciana*, respectively. *Avena ludoviciana* had fewer tillers than the other two species. The plants emerged at 37.58 GDD at the soil surface temperature, which corresponds to 7 days after sowing. The growing cycles of the species differed: 196 days for *A*. *sterilis*, 201 days for *A*. *fatua*, and 209 days for *A*. *ludoviciana* after emergence, although there were no clear differences in earlier growth stages. This study provides initial basic information about the *Avena* spp., and it is concluded that even if a field has mixed *Avena* populations, herbicides can be applied simultaneously because the early development stages of the three species are very similar. In future prospects, there is a need for proper studies about the management of all *Avena* spp. on the basis of growth stages and growing degree days in regional context.

## Introduction

*Avena* species are among the most important weeds worldwide [1] due to their ability to occupy complex habitats via genetic flexibility under climate change scenario [2]. Among the nine weedy *Avena* species, *A*. *fatua* (AVEFA), *A*. *sterilis* (AVEST) and *A*. *ludoviciana* (AVELU) have the largest distribution as compared with other *Avena* spp. and are distributed in more countries [3]. In Australia, AVEFA and AVELU are found together in several fields [4]. In Türkiye, *Avena* species became more important weeds in winter cereals in several regions of the country during the 1980s [5]. AVEFA, AVEST and AVELU can be found together in a field, and combinations of any two species are more common. *Avena ludoviciana* (this name will be used in the text) is accepted as a subspecies of *A*. *sterilis* by many authorities [6, 7].

*Avena* species cause yield and quality losses in many crops, especially in winter cereals, and lead to decrease in biodiversity [8–16]. The foremost method for controlling *Avena* species is the use of herbicide, but their extensive use causes herbicide resistance [9]. Herbicide-resistant *Avena* species have caused problems that are becoming more complex in Türkiye as well as in other countries [17–20].

Successful and sustainable control of *Avena* species requires increased knowledge of their ecology and biology as well as accurate prediction of growth stages [21–25]. Previously germination and emergence studies included the effects of temperature, water potential, dormancy, and burial depth, but conflicting results were observed [22]. AVEFAs were able to germinate at a much greater percentage and at deeper levels in greenhouses than in fields [26]. It showed better emergence at the 2–8 cm depth than at deeper depths. A large quantity of seeds was also emerged from deeper depths, although germination rate was higher at shallower depths [22]. The varying dormancy patterns among the AVEFA accessions were attributed to location and season [24]. The germination temperature for AVEST in petri dish experiments was observed and 2°C was the minimum temperature, 30°C was the maximum temperature while the optimum temperature was 10°C [27]. The emergence temperatures for AVELU were reported to be -1°C as the base temperature, 5.8°C as the optimum temperature and 18°C as the maximum temperature [28]. AVEST and AVEFA germinated at 5–30°C and osmotic potentials of -25 to -1400 KPa, but AVEST germinated and emerged better at 10°C and above 20°C [29]. In another study with AVEFA, neither germination nor emergence occurred at 32°C, but germination was greater between 10–21°C, and germination was slower at 10°C than at higher temperatures [22]. The field capacity was not allowed for the emergence of AVEFA, but between 50 and 75% of the field capacity was the best for emergence [22].

The growth characteristics of *Avena* spp. have also been widely studied. The AVEFA accessions showed variation in plant height, number of tillers, days to panicle emergence, grain yield, and response to certain herbicides, such as diclofop and flamprop [24]. In another study, variations in the tiller and seed numbers of AVEFA between years were detected, although these differences were attributed to the greater precipitation that caused greater numbers of plants. However, there was also a difference among individuals [26]. The plant height and number of leaves per plant of the AVEFA increased almost linearly during the first 6 weeks after emergence, and tillering occurred mainly between the 2^nd^ and 4^th^ weeks after emergence; however, the dry weight accumulation was slow in the first three weeks and then increased until the 8^th^ week [23]. The optimum temperature for growth was 20/12°C day/night, although at 28–20°C plants performed better in the initial growth stages [23]. Low fertilizer amounts and light intensities caused lower levels of growth parameters, and the photoperiod also affected AVEFA growth [23].

To date, the development stages of *Avena* species, including their connection with emergence times have been studied for more than 100 years. However, important weedy *Avena* species have not been compared in common garden experiments, although some studies have compared two of them. The present study aimed to investigate the differences in growth stages among three common *Avena* species through a common field experiment.

## Materials and methods

### Seed source

Seeds from six different populations were collected from wheat fields in different parts of Turkey during June 2013. These seeds were collected from Mediterranean, Aegean and Southeast Anatolia region as these are the major region of Turkey and represent whole country. The author confirms that permission for seed collection was taken from the respective farmers and study did not involve protected or endangered plant species, hence, there was no need to take permission from specific authority. Seeds were cleaned and kept in a refrigerator at 4°C until use (Table 1). All populations were used in all the experiments.

**Table 1:** Populations and their features.

Population Code	*Avena* Species	Location (Province)	Location (Region)
013 ADA 02	*A*. *sterilis* subsp *sterilis*	Adana	Mediterranean
013 ADA 03	*A*. *sterilis* subsp *sterilis*	Adana	Mediterranean
013 IZM 01	*A*. *fatua*	Izmir	Aegean
013 DİY 04	*A*. *fatua*	Diyarbakir	Southeast Anatolia
013 ADA 01	*A*. *sterilis* subsp *ludoviciana*	Adana	Mediterranean

### Germination experiments

Germination experiments were performed to determine whether there was a difference in germination patterns among the three *Avena* species. The six populations mentioned earlier were used and experiment was conducted using completely randomized design (CRD). Four replications were performed in an incubator adjusted to 10°C according to the study [29] on 10 November 2014 and the same pattern was repeated afterward. Seeds were kept in 5% NaClO solution for 30 seconds to sterilize the seed surfaces, followed by rinsing twice with distilled water for 60 seconds. For each Petri dish (90 mm in diameter), 15 seeds were placed into two layers of Whatman No: 1 filter paper, and then 5 ml of distilled water with 0.01% KNO3 was added. Germinated seeds whose radicles became longer than the diameter of the seeds was counted and removed from the Petri dishes daily. The experiment terminated on the 15^th^ day, and the remaining ungerminated seeds were subjected to the triphenil tetrazolium chloride test (TTC) using 0.01% TTC solution. The seeds were kept at 40°C for one and a half hours, and seeds with red color were considered alive.

### Growth experiment of *Avena* species

A screen house experiment was conducted in the Plant Protection Department, Faculty of Agriculture, Ege University, Bornova, Izmir (38° 27′ 26″ N and 27° 13′ 47″ E) using all six populations. The experimental area was disinfected to avoid any additional species interference. Small plots of one square meter were established and fertilized similarly to common farmer practices in the wheat field in the region i.e., NPK 150–50–60 kg/ha. Temporary screen houses were established on plots using 40 meshes of screen material. *Avena* seeds were sown at a 10 cm inter-row distance on 12 November 2014, and the soil was good for wheat sowing, as farmers have done to simulate growth in a wheat field. During the observation period, other weed species were removed. The growth stages were recorded daily according to the BBCH scale [30]. As soon as the first ten plants in all plots emerged (BBCH 10), these plants were marked in each plot to follow the growth and other parameters.

After the flag leaf opened, SPAD values were measured from the flag leaf and leaf before the flag leaf on 10 April 2015. On the same day, the width and ligule length of the leaf before the flag leaf were measured.

Although the observed species did not mature on the same date, all the populations were harvested by cutting them from the soil on 25.05.2015. After separating 10 seeds with or without awn, the plant height was measured, and the number of tillers was counted. The harvested plants were subsequently placed in a dryer at 65°C for 48 hours and their dry weight was recorded using measuring scale.

Growth data were explained using growing degree days (GDD) as well as calendar days. The GDD for emergence was calculated according to [31]:

If the soil temperature was between Tb and To, then,

DTD = T − Tb

If the soil temperature was between To and Tc

DTD = (T − Tb)*[1 − (T − Tb)/(Tc − Tb)]

and if T < Tb or T > Tc, then TT = 0

where T is the mean daily temperature; Tb is the base temperature; To is the optimum temperature; and Tc is the maximum temperature; and Tb = −1.0, To = 5.8, and Tc = 18.0 C for AVELU (31). In other published studies, different values of Tb = 2, T0 = 10 and Tc = 30 were found for AVEST (27); moreover, Tb = 2.2 was found for AVEFU [32].

For the growth equation, DTD = T–Tb was used. In this case, Tb was set to 0, as in [33].

The data were subjected to ANOVA and descriptive statistics using R software (R Core Team, 2023) [34, 35]. Correlation analysis was also done for comparing all recorded parameters.

## Results

There was no statistically significant difference in germination among the populations. All three species germinated within seven days after sowing and the germination rate ranged from 97–100% (median = 100.00%; average = 99.50%), and the aliveness of the seeds ranged from 88–100% (median = 95.50%; average = 94.67%).

**Fig 1:**
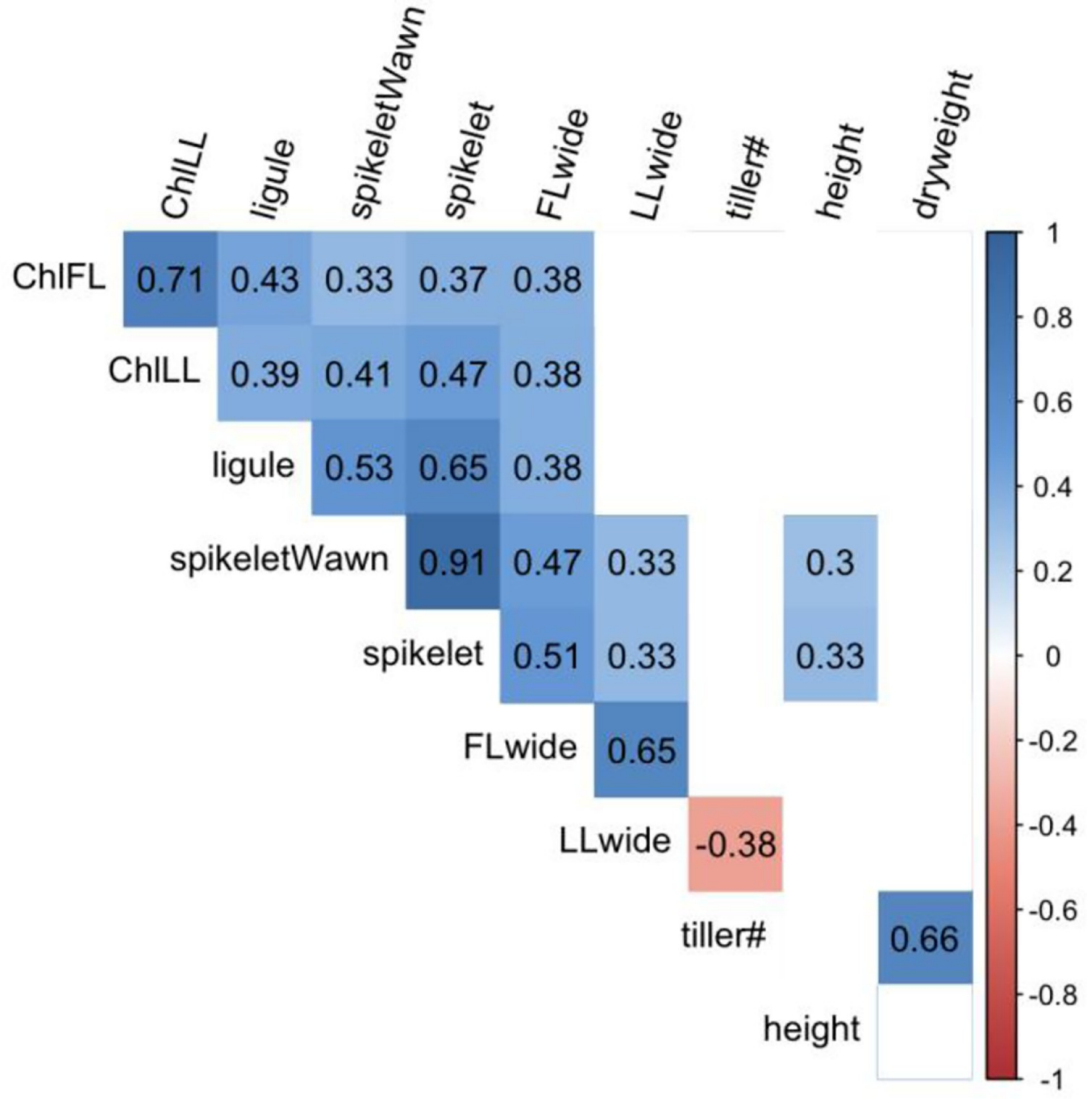
Correlations among the measured parameters (ChlFL, SPAD values of flag leaf; ChlLL, SPAD values of leaf before flag leaf; SpikeletWawn, Spikelet with awn; FLwide, width of flag leaf; LLwide, width of leaf before flag leaf).

The height of the spikelet with awn and the spikelet itself showed the highest correlation value ranging between 0.91 and 0.51 respectively. The SPAD values and width of the flag leaf and the leaf before the flag leaf were strongly correlated and were 0.71 and 0.65, respectively. The number of tillers and the dry weight of the plants also exhibited a greater correlation (0.66). Lower positive correlations were found for spikelet (with and without awn) and width of flag leaf, which were 0.47 and 0.51, respectively. Ligule length was the correlated length of the spikelet with awn (0.53) and without awn (0.65). There was only a slight negative correlation between the number of tillers and the number of leaves before the flag leaf (-0.38) (Fig 1).

**Fig 2:**
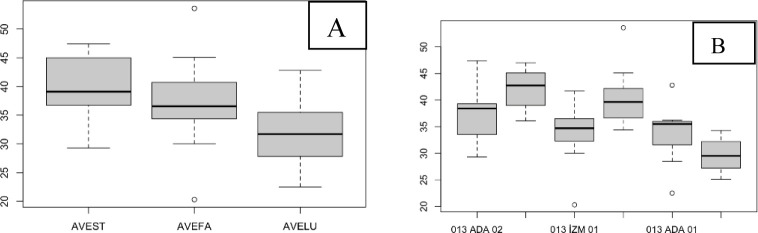
The SPAD values of the flag leaves. (A), species level; (B), population level.

The SPAD value of the flag leaves did not significantly differ among the species, although AVELU generally had lower SPAD values (Fig 2A). Populations ada03 and diy04 had higher SPAD values and were significantly different from diy03, which had lower SPAD values (Fig 2B; Table 2).

**Table 2:** Mean values of parameters at the species and population levels.

Species	AVEST			AVEFA			AVELU		
Parameters	Species	Ada02	Ada03	Species	İzm01	Diy04	Species	Ada01	Diy03
SPAD values of flag leaf	39.96	37.99	41.93	37.09	33.54	40.63	31.59	33.63	29.55
SPAD values of leaf before flag leaf	38.53	37.25	39.82	33.55	32.08	35.03	33.55	35.60	29.23
The length of ligule	9.149	7.521	10.78	5.362	5.427	5.298	4.564	5.720	3.407
The length of spikelet with awn	81.47	80.63	82.31	49.39	48.23	50.55	48.88	49.20	48.57
The length of spikelet without awn	37.84	37.22	38.46	25.64	24.95	26.32	26.81	27.48	26.15
The width of flag leaf	19.91	18.70	21.11	14.30	13.67	14.94	13.82	14.43	13.22
The width of leaf before flag leaf	22.47	21.32	23.62	16.90	16.94	16.87	16.90	22.52	20.28
The number of tillers	9.95	11.30	8.60	15.20	13.2	17.20	6.15	6.70	5.60
Plant height	201.8	205.7	198.0	186.1	181.1	191.1	189.9	202.0	177.8
Dry weight	73.03	83.69	62.37	73.29	50.81	95.78	43.05	49.69	36.42

**Fig 3:**
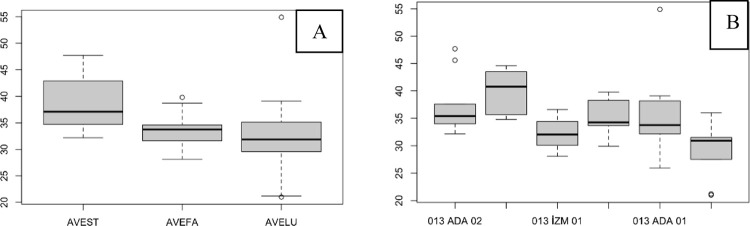
The SPAD values of the leaf before the flag leaf. (A), species level; (B), population level.

There was no significant difference among the species in terms of the SPAD values of the leaf before the flag leaf, although AVEST mostly had higher SPAD values and AVELU had lower SPAD values (Fig 3A). Similar differences were observed between populations ada03 and diy03 (Fig 3B; Table 2). In addition, the SPAD value of population izm01 was significantly different from that of ada03 before the flag leaf was removed (Fig 3B).

**Fig 4:**
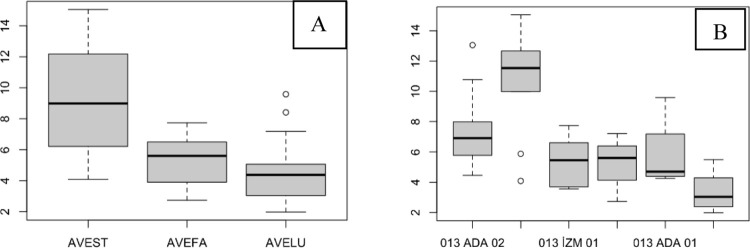
The length of spikelets with awns and without awns of AVESP. (A), species level with awn; (B), population level with awn.

The length of the ligules on AVEST was greater in general, and ada03 had significantly greater ligule lengths than did the other populations (Fig 4; Table 2).

**Fig 5:**
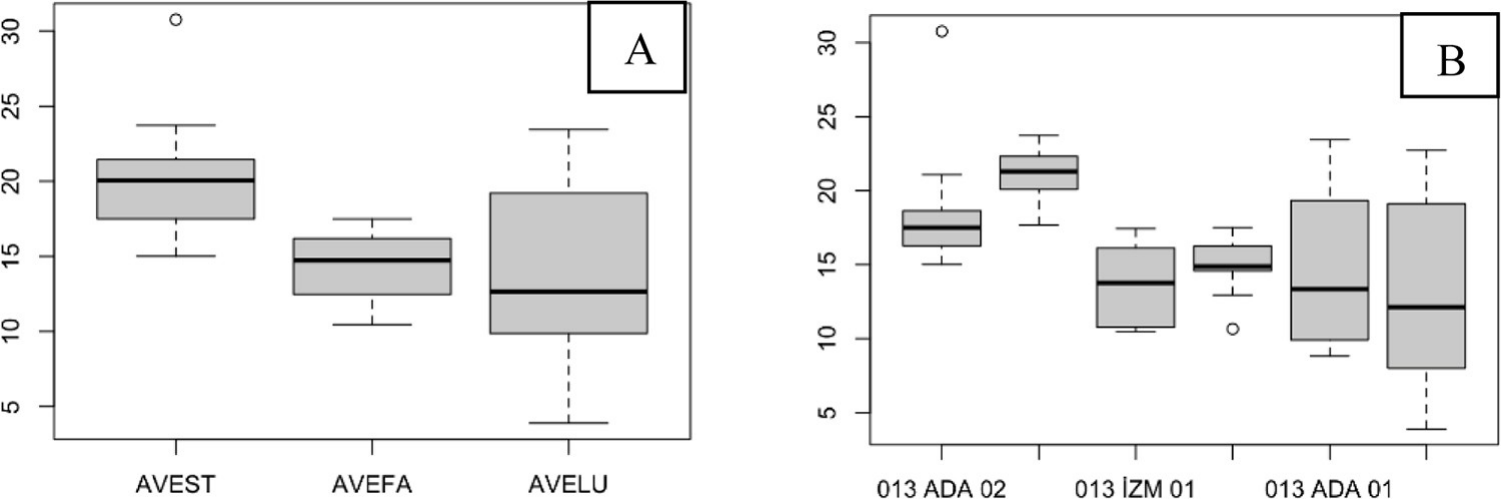
The width of the flag leaf of AVESP. (A), species level; (B), population level.

The width of the flag leaf exhibited high variation among individual populations (Fig 5A). There was no significant difference among the species or populations, although the average width was greater for AVEST (Fig 5B; Table 2).

**Fig 6:**
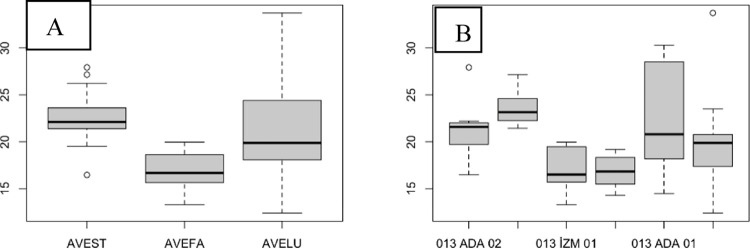
The width of the leaf before the flag leaf of AVESP. (A), species level; (B), population level.

Although the AVEFA had lower width values, there was no significant difference among the species or populations (Fig 6A; Table 2). There was considerable variation in the population of 013ada01 individuals (Fig 6B).

**Fig 7:**
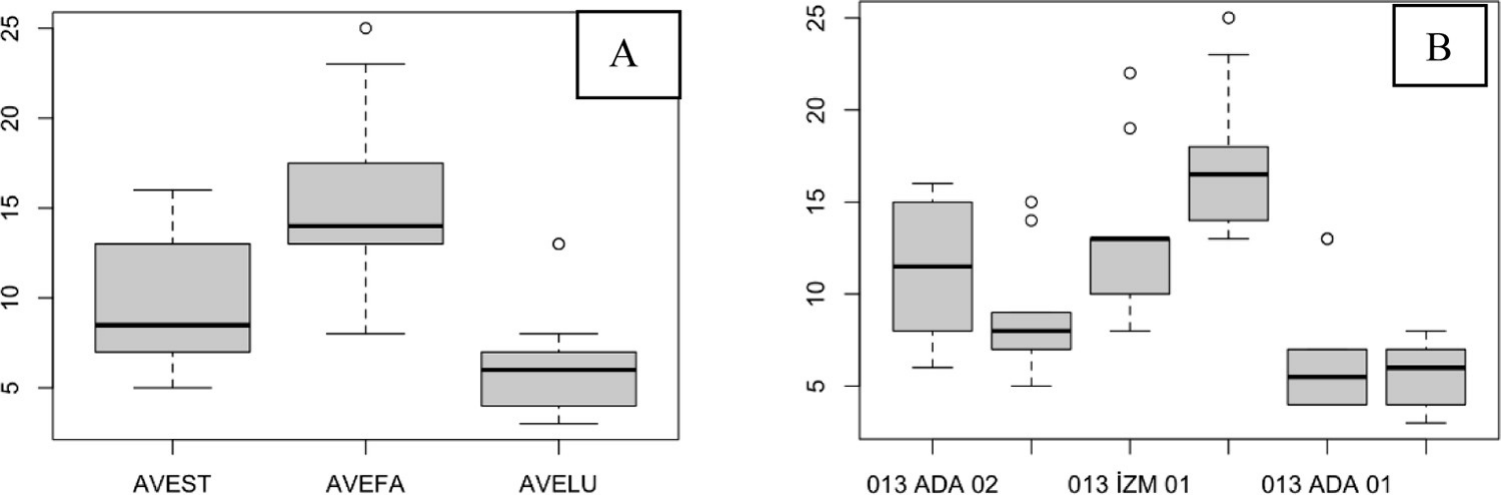
The number of tillers of AVESP. (A), species level; (B), population level.

AVEFA had the highest number of tillers (mean 15.20, minimum 8.00, maximum 25.00), while AVELU had the lowest tiller numbers (mean 6.15, minimum 3.00, maximum 13.00) (Fig 7A; Table 2). The average number of tillers of AVESTs was 9.95 (min 5.00, max 16.00). The population 013ada03 from the AVEST was similar to both AVELU population (Fig 7B). However, only the AVELU populations were significantly different from the AVEFA populations.

**Fig 8:**
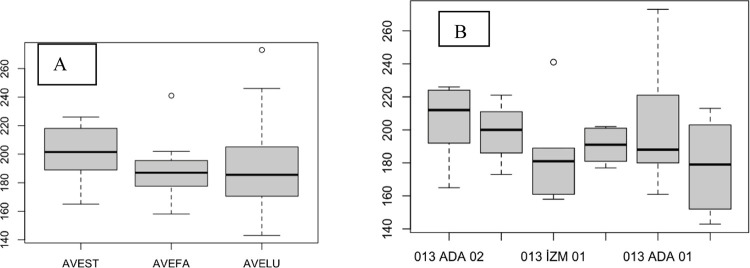
Plant height of AVESP. (A), species level; (B), population level.

The plant height did not differ between species or at the population level, but the AVELU populations exhibited greater intrapopulation variation (Fig 8(A), 8(B); Table 2). The tallest and shortest individuals were in the AVELU group.

**Fig 9:**
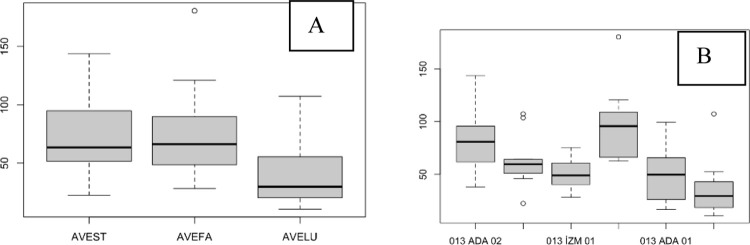
Plant dry weight of AVESP. (A), species level; (B), population level.

Species did not differ in dry weight (Fig 9A), but two populations, Diy04 and Diy03, were significantly different (Fig 9B).

One week after sowing (19 Nov), all the populations were observed at the soil surface, for which the GDDacc was 32.37 (Table 3). A day later, the first true leaf emerged only at population diy03, and the following day, all the populations had a true leaf. In addition, the first true leaf unfolded only at pop ada02.

**Table 3:** Emergence of seeds (BBCH scale) related to calendar days and accumulated GDD.

	Accumulated GDD	A.S.		A.F.		A.L.	
		013 ADA 02	013 ADA 03	013 İZM 01	013 DİY 04	013 ADA 01	013 DİY 03
12.11.2014	4.45	0	0	0	0	0	0
19.11.2014	32.87	9	9	9	9	9	9
20.11.2014	37.58	9	9	9	9	9	10

**Fig 10:**
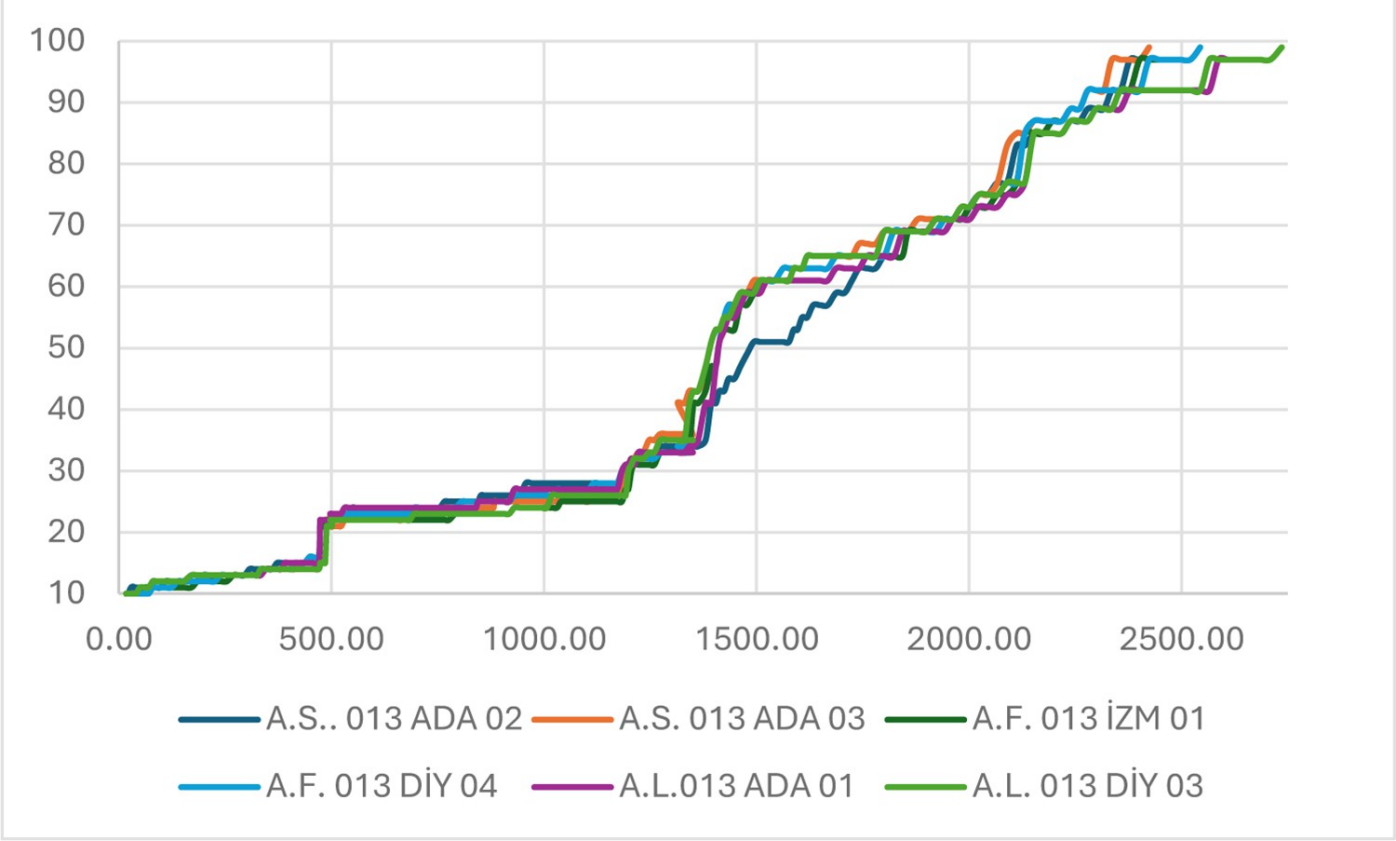
Developmental stages of AVESP populations and their relationships with accumulated GDD.

The development stages and their relationships with GDDacc are shown in Fig 10, and their starting dates are given in Table 4. All the populations developed five leaves except diy04, which had 6 leaves. The tillering stage started on 30 December with GDDacc 473.52 in four populations with one or two tillers. Tillering started four days later for the Ada03 and diy03 populations at GDDacc 489.05. The populations had different number of tillers, as follows: ada02 and diy04 eight, diy03 six and the remaining populations had seven tillers. Stem elongation started on 17 March (GDDacc 1183.45) for three populations, and the other populations exhibited one-day differences: ada03 on 18 March, diy03 on 19 March and izm01 on 20 March (GDD acc 1205.80). Up to six nodes were visible (ada03), the lowest was four (izm01), and the remaining had five nodes. Booting started between 01 April (ada03) and 07 April (ada02) and between these two AVESTs in the other populations. The first heading occurred on diy03 on 07 April (GDDacc 1393.80), followed by four other heading events on 09 April (GDDacc 1412.85) and ada02 15 April (GDDacc 1493.70). The first flowering population was ada03 on 15 April (GDDacc 1493.70), which was immediately followed by other populations except ada02, which was on 30 April (GDDacc 1724.65). Ripening started between 8 and 12 May, and all populations reached the dough stage between 19 and 21 May. AVEST completed its life cycle on 03 June (GDDacc 2423.82), AVEFA on 8 June (GDDacc 2543.97) and AVELU at 16 June (GDDacc 2735.72).

**Table 4:** Starting dates of main development stages of populations.

		A.S. 013 ADA 02	A.S. 013 ADA 03	A.F. 013 İZM 01	A.F. 013 DİY 04	A.L. 013 ADA 01	A.L. 013 DİY 03
Coleoptile breaks soil (emergence)	Calendar Days	19.Nov.2014	19.Nov.2014	19.Nov.2014	19.Nov.2014	19.11Nov.2014	19.Nov.2014
	Accumulated GDD	0	0	0	0	0	0
The first leaf emerged	Calendar Days	21.Nov.2014	21.Nov.2014	21.Nov.2014	21.Nov.2014	21.Nov.2014	20.Nov.2014
	Accumulated GDD	28.90	28.90	28.90	28.90	28.90	17.10
Tillering	Calendar Days	30Dec	03Jan	30Dec	30Dec	30Dec	03Jan
	Accumulated GDD	473.52	489.05	473.52	473.52	473.52	489.05
Stem elongation	Calendar Days	17Mar	18Mar	20Mar	17Mar	17.Mar	19Mar
	Accumulated GDD	1183.45	1192.25	1205.80	1183.45	1183.45	1198.70
Booting	Calendar Days	07Apr	01Apr	04.Apr	06.Apr	06.Apr	03.Apr
	Accumulated GDD	1393.80	1314.90	1352.05	1380.20	1380.20	1342.05
Heading	Calendar Days	15Apr	09.04.2015	09.04.2015	09.04.2015	09.04.2015	07Apr
	Accumulated GDD	1493.70	1412.85	1412.85	1412.85	1412.85	1393.80
Flowering	Calendar Days	30Apr	15Apr	16Apr	16Apr	17 Apr	16Apr
	Accumulated GDD	1724.65	1493.70	1508.65	1508.65	1524.35	1508.65
Watery ripe	Calendar Days	12May	08May	12.May	11.May	12.May	10.May
	Accumulated GDD	1962.15	1879.40	1962.15	1942.75	1962.15	1922.25
Dough	Calendar Days	21.May	19.May	20.May	20.May	21.May	21.May
	Accumulated GDD	2152.75	2111.90	2131.55	2131.55	2152.75	2152.75

## Discussion

All the populations emerged seven days after sowing, similar to the previous study which had reported the emergence of *Avena* spp. in 7–10 days [24]. The reason for the uniformity in germination is their ability to adopt a wide range of environmental conditions [12]. Also, the seed viability and persistence are widely dependent on environmental conditions soil factors [24]. *Avena* spp. are capable of germinating at a temperature range of 5–30°C and -0.025–1.4 MPa solute potential [29]. Tillering started 41 DAE, but two populations AVEST and AVELU (13ada03 and 13diyo3) were 4 days late. Tillering lasted 77 (74–80) days [22], which found that 2–4 weeks after emergence was the main tillering time for AVEFA; however, in another study, tillering started at 32^nd^ DAE and completed 70^th^ to 84^th^ DAE regarding the emergence time of cohorts [25]. However, the accumulated GDD difference were only 15.53. The reason for a slight change in tillering duration is that the origin of these populations, as population from southern grain region produced tillers 7–8 days earlier as compared with northern grain region of Australia [36]. Similarly, the beginning of stem elongation occurred between 17 and 20 March (118–121 days after emergence); again, the difference in accumulated GDD was 22.35 only among the populations. Booting started on 01 April (133 days after emergence) for ada03, and the latest one was ada02 on 07 April; there was a greater difference (GDDacc 78.9) between these AVEST populations. Ada02 was also the most retarded population during heading and flowering, and the difference in accumulated GDD was greater than that in the earliest populations (99.9 and 230.95, respectively). Retardation of ada02 cells continued in the early stages of maturation, but the difference in accumulated GDD decreased (82.75). The fastest population was mostly the other AVEST, ada03. Even in the dough stage, there were two days of retardation, during which the difference in accumulated GDD decreased. The end of the life cycle of these two AVEST populations was the same at 196 days. AVEFA completed its life cycle 201 days after emergence, and AVEFU completed its life cycle 209 days after emergence. The variability in these parameters and differences in the life cycle is strongly correlated with the soil conditions and environmental factors [2, 24]. All these parameters are helpful for adopting management strategies for these spp. For example, previous data showed that December will be a good time to apply herbicides because early applications (1–2 leaf stages) to control AVEFAs causes higher wheat yields even though herbicides are more effective in the tillering stage [36]. Early applications of herbicides to control *Avena* species were also recommended [37]. On the other hand, AVELU emergence flush occurs between November and February under Mediterranean conditions (28). The ability to catch later flush herbicide applications can be delayed until early spring. Even if a field has mixed *Avena* populations, herbicides can be applied at the same time because the early development stages are very similar among the three species. In addition, there was a difference in phenology between the southern and northern sides of Spain for AVELU, where tillering occurred earlier in southern sites, between November 25 and the end of December, but it occurred at the end of January. In both these regions, both temperature and rainfall pattern are different so there is a change in biology of these species in order to adapt to these conditions [12]. The experimental site in Bornova was located between Spain and parallels, where tillering started in late December and early January, which is in agreement with the findings of the Spain study. Tillering started at 48 days after sowing in Spain and Bornova, but it lasted longer in Bornova, up to 104-110^th^ day for AVELU, 106-123^rd^ day for AVEFA, and 117-125^th^ day for AVEST after sowing, which was the 79^th^ day in Spain.

The SPAD values were not significantly differed among species, but the AVELU generally had lower SPAD values. The reason for the decline in SPAD values was the difference between study sites and species type. Previously it has been observed that the SPAD values were strongly affected by the location of the study site [38]. This difference significantly influences competitive interactions with crop plants and overall weed management strategies.

There were differences between two populations of the same *Avena* species in many features in the present study. According to reference [24], among 302 AVEFA accessions, tillers per plant ranged from 10 to 42, with an average of 21 to 31 tillers per plant. There were five leaves on the main stem (except for diy03 of AVEFA, which had 6 leaves), and the number of tillers was 15 for AVEFA, 10 for AVEST, and 6 for AVELU; these numbers are greater than those in an earlier study in which AVEFA had 3–4 leaves and 10–15 tillers [23]. In a field study in which 25 individuals were selected each year, the average number of AVEFA tillers was 14 and 24 per plant in the first and second years of the experiment, respectively [26]. In addition, the tiller number per plant varied between 11 and 39, which was 8–25 for AVEFA in the present study.

The average plant height was approximately 2 m, which is roughly double the values reported in earlier studies in which the maximum height of the AVEFA was 79 cm [26] and 65 to 95 cm [25]. The duration of flag leaf emergence exceeded 130 days for all populations, and heading occurred 3–8 days after booting, whereas the duration of booting started was 42–51 DAE, and heading started one week later in an earlier study [23]. The difference between [25] and the current study is the time of the experiments, which were spring and winter growth experiments. In addition, the flora of Turkey was 30–130 (-150) cm for AVEST and AVELU and 45–80 (-150) cm for AVEFA [6]. In an experiment with AVEFA accessions, height ranged from 61 to 148 cm, but the average height was 90 to 118 cm [24].

The length of the spikelets with awn or without awn for AVEST was significantly different from that for AVEFA and AVELU. The Flora of Turkey has spikelet lengths of 22–27 mm for AVEFA, 20–30 mm for AVELU and 30–45 mm for AVEST, which supports the results of current study. Ligule length for AVEFA was 5.36 mm (2.73–7.73 mm), that for AVELU was 4.56 mm (1.97–9.58 mm), and that for AVEST was 9.15 mm (4.08–15.05 mm) in the experiment, but the flora of Turkey had 4–6 mm for AVEFA and 3–8 mm for AVEST and AVELU without discriminating subspecies [6]. Although there was no clear cutoff point for identifying species in our study, any individual with more than 10 mm of ligule was identified as having AVEST (i.e., *A*. *sterilis* subsp *sterilis*). This partly helps differentiate species in earlier stages. The flagellum of Turkey can be lengthened with articulation to allow more precise differentiation of these three species.

Apart from phenology, some ecological features are also helpful in determining the specific *Avena* species. For example, germination of *A*. *ludoviciana* is grown in temperate zone and favored more by low temperature as compared with the *A*. *fatua* [12]. Moreover, *A*. *ludoviciana* has a wider ability to adapt drought conditions than *A*. *fatua* [29]. These variations in species could be helpful in determining the specific species and adopting a methodological approach to control these species. Moreover, studying these factors could be helpful in determining the dynamics of these species in different cropping systems.

## Conclusion

In the current study, growth cycles of three *Avena* species have been studied. There were three significant differences among the studied species. The spikelet length, which is currently used to distinguish AVEST from AVELU; the ligule length, which is suggested for partial differentiation; and the length of the growing cycle, which is considered to be 196 days for AVEST, 201 days after emergence for AVEFA, and 209 days for AVELU.

## Supporting information

S1 Raw data (XLSX)
